# Influence of Different Heater Structures on the Temperature Field of AlN Crystal Growth by Resistance Heating

**DOI:** 10.3390/ma14237441

**Published:** 2021-12-04

**Authors:** Ruixian Yu, Chengmin Chen, Guodong Wang, Guangxia Liu, Shouzhi Wang, Xiaobo Hu, Ma Lei, Xiangang Xu, Lei Zhang

**Affiliations:** 1 State Key Laboratory of Crystal Materials, Institute of Novel Semiconductors, Shandong University, Jinan 250100, China; yuruixian0001@126.com (R.Y.); wangshouzhi6@163.com (S.W.); xxu@sdu.edu.cn (X.X.); 2 Energy Institute, Qilu University of Technology (Shandong Academy of Sciences), Jinan 250100, China; cm_chen1989@163.com (C.C.); liugx@sderi.cn (G.L.); 3 Jinan Institute of Supercomputing Technology, Jinan 250100, China; 4 School of Opto-Electronic Engineering, Zaozhuang University, Zaozhuang 277160, China; leima_1017@163.com

**Keywords:** AlN crystal, temperature field, crystal growth, numerical simulation

## Abstract

Based on the actual hot zone structure of an AlN crystal growth resistance furnace, the global numerical simulation on the heat transfer process in the AlN crystal growth was performed. The influence of different heater structures on the growth of AlN crystals was investigated. It was found that the top heater can effectively reduce the axial temperature gradient, and the side heater 2 has a similar effect on the axial gradient, but the effect feedback is slightly weaker. The axial temperature gradient tends to increase when the bottom heater is added to the furnace, and the adjustable range of the axial temperature gradient of the side 1 heater + bottom heater mode is the largest. Our work will provide important reference values for AlN crystal growth by the resistance method.

## 1. Introduction

In recent years, ultra-wide bandgap semiconductor materials represented by aluminum nitride (AlN) have been widely used in different fields due to their excellent high-frequency power characteristics, stable high-temperature performance, low energy loss, and good UV transmittance [1,2,3]. Therefore, it has great application prospects in the fields of high-efficiency optoelectronic devices, high-power high-frequency electronic devices, ultra-high voltage power electronic devices, deep ultraviolet warning and guidance, and deep ultraviolet light-emitting diode (DUV LED) disinfection [4,5,6,7,8]. At the same time, AlN crystal is also an ideal substrate material for the Al-rich epitaxial growth of group III nitrides [9]. Especially in the field of power electronics, AlN has a very high critical breakdown electric field, and the fabricated power devices have high off-state blocking voltage, ultra-low on-resistance, and ultra-fast switching time. The comprehensive performance is 10–15 times that of SiC and GaN power devices. So far, a variety of methods have been developed to prepare AlN crystals, which mainly include hydride vapor phase epitaxy (HVPE) [3], molecular beam epitaxy (MBE), metal organic compound vapor deposition (MOCVD), solution growth, physical vapor transport (PVT) [2,10], and so on. The PVT method has the advantages of a simple growth process, fast growth rate, low dislocation density, good crystal integrity, and high safety, and has been proven to be one of the most effective methods for the preparation of AlN bulk single crystals [10,11,12,13,14]. In recent years, AlN crystal growth has mainly focused on the following three strategies [2,10]: (1) spontaneous nucleation, (2) homoepitaxial growth on AlN substrates, and (3) heteroepitaxial growth on SiC substrates. For spontaneous nucleation growth, the AlN extension angle is small (10–15°), and it is difficult and time-consuming to extend the diameter. The lack of large-size AlN single crystal substrates limits the development of AlN seed crystal homogeneous growth technology. An SiC substrate can be used as a seed to provide the possibility to obtain large-size AlN crystals. However, there are often cracks in the AlN single crystals grown on SiC seeds. The concentration of Si and C impurities seriously affects the performance of the device. Regardless of the strategy, the use of AlN seeds for homoepitaxial growth is the ultimate way to obtain high-quality, large-sized AlN crystals. For different growth strategies, thermal field materials, thermal field design, and growth processes are the core for AlN crystal growth. Since the growth temperature of AlN crystals is usually 1800–2350 °C, the maintenance of a stable growth environment for a long time under extreme high-temperature conditions, the selection of thermal field materials, and the design of thermal field structures are particularly important.

At present, there are two growth strategies commonly used to prepare AlN crystals by the PVT method, including (1) radio frequency induction heating + a graphite insulation system and (2) resistance heating + a tungsten molybdenum insulation system. The radio frequency induction heating + graphite insulation system uses graphite felt as insulation material. It is reported that C impurity usually exists in the form of CN in AlN crystals, and thus it is easy to form an ultraviolet absorption peak at about 265 nm (4.7 eV) [15]. The resistance heating + tungsten and molybdenum insulation system has very little C impurity content, and the O impurity content can be further reduced by improving the raw material sintering process, so it is easy to grow AlN crystals with high UV transmittance.

Q. K. Wang et al. simulated the influence of thermal stress and crucible shape on the AlN crystal quality and the transport of vapor species for a resistance heating furnace and found that the distribution and magnitude of stress in AlN crystals are closely related to the temperature gradient and growth direction of the growing crystal [15,16]. The parasitic polycrystalline grains around the crystal are reduced and suppressed by designing different conical confinement rings. Z.H. Wang et al. used FEMAG software to perform a global quasi-steady-state numerical simulation of the AlN crystal growth thermal field [17]. The influences of the size of the induction heater, the thickness of the crucible, the tungsten heat shield, etc. on the thermal field were simulated. Z. Y. Qin et al. investigated the relationship between the hot zone structure and the temperature distribution of the growth chamber [18]. The simulation results showed that the thickness of the crucible, the size of the induction heating furnace, and the number of tungsten heat shields have a great influence on the temperature gradient. Y. Yu et al. investigated the effect of adding a tungsten sink to the top of the AlN growth crucible on the temperature filed. This structure makes the radial temperature gradient on the growth cavity uniform [19].

Although researchers have performed a lot of simulation work on the thermal field and thermal stress for PVT AlN crystal growth, there are no reports on the influence of different heater structures on the growth of AlN crystals by the resistance method. In this paper, the COMSOL Multiphysics software is used to simulate the thermal field of the self-designed resistance method AlN crystal growth furnace under different heater structures. The optimal heater structure suitable for AlN crystal growth is obtained, which provides a theoretical basis for the design and parameter optimization of the hot zone structure for an AlN crystal growth resistance furnace.

## 2. Simulation Model and Physical Parameters

### 2.1. Subsection Physical Model and Physical Parameters

Based on the actual hot zone structure and crystal growth process parameters, the finite element method is used to perform global numerical simulation on the heat transfer process in the crystal growth. The calculation model couples all structural units in the crystal growth furnace, so that the global temperature distribution and gas flow in the entire growth furnace can be accurately predicted. For the energy control equation, continuity equation, fluid momentum control equation, etc. used by COMSOL software 5.3, refer to related literature [20,21,22]. The physical parameters of the materials used in the simulation are shown in Table 1.

It should be noted that (1) the quasi-steady-state model is used to carry out the global calculation of the thermal field in the growth furnace. (2) A large number of studies have shown that the main heat transfer types in the high-temperature growth furnace are radiation heat transfer and heat transfer (especially radiation heat transfer). However, the effect of convective heat transfer on the thermal field in the furnace is very limited [23,24]. Therefore, all calculations in this paper ignore the influence of gas flow in the growth cavity. (3) It is considered that the heater itself has no heat consumption, and all the heat is consumed in the heating system. (4) It is assumed that the outermost temperature of the system is constant (300 K).

### 2.2. Thermal Field Geometric Model and Simulation Parameters

**Figure 1:**
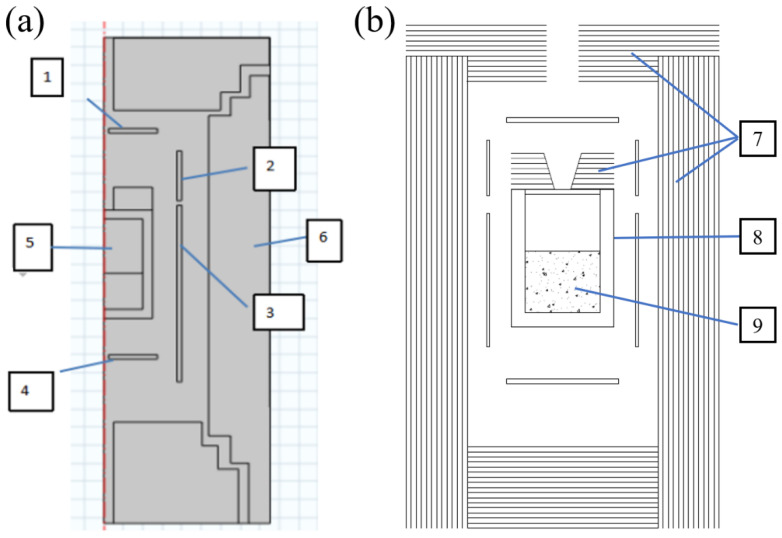
(**a**) Schematic diagram of different heater structures for AlN crystal growth resistance furnace. (**b**) Schematic diagram of tungsten and molybdenum thermal field structure for AlN crystal growth resistance furnace. 1—Top heater; 2—side heater 2; 3—side heater 1; 4—bottom heater; 5—growth chamber; 6 and 7—tungsten and molybdenum insulation screen; 8—tungsten crucible; 9—AlN raw materials.

Figure 1 is a schematic diagram of a home-made AlN crystal growth furnace by the PVT method. The furnace is mainly composed of a resistance heater, a multi-layer high-purity tungsten screen, and a high-purity tungsten crucible. In order to flexibly adjust the thermal field, the upper, lower, and side heater structures are designed. The influences of different heaters (side heating mode, side heater + bottom heater mode, side heater + top heater mode, side 1 heater + side 2 heater mode) on the thermal field in the tungsten crucible and the growth of AlN crystals are investigated by numerical simulation. In the simulation process, the inner diameter of the tungsten crucible is set to 60 mm, and the pre-sintered raw material is placed at the bottom of the crucible, with the size set to 56 mm × 40 mm. The crucible lid can be placed on the seed, tungsten sheet, or other substrates according to the process requirements. In the simulation process, the insulation materials are the same, the assembly conditions are the same, and the crucible is set to a constant pressure of 50 kPa of high-purity nitrogen atmosphere. The material property parameters used in all the calculations can be referred to in the literature [13,17,19].

## 3. Simulation Results and Discussion

In order to obtain high-quality AlN crystals, a reasonable thermal field distribution must be established in the crystal growth system. In the process of crystal growth by the PVT method, the inflow and outflow heat of the crystal growth interface should reach thermal equilibrium, as shown in Equation (1):

Q_outflow_ = Q_inflow_ = Q_1_ + Q_2_ + Q_3_ + …… (1)

The Q_outflow_ is the heat that flows out from the crystal growth interface through the grown crystal, and the Q_inflow_ is the heat flowing into the crystal growth interface. Q_1_ is the heat of the growth species flowing into the growth interface. Q_2_ is the heat of radiation flowing into the crystal growth interface during the growth process. Q_3_ is the latent heat of crystallization. The conditions for the stable growth of crystals in the PVT method is that the heat of the growth cavity gas flowing into the growth interface is positive (Q_1_ > 0) and cannot be too large. If the above conditions are not met, when Q_1_ < 0, there will be defects such as dendrites, polycrystalline, etc. If Q_1_ is too large, polycrystalline defects will appear in the crystal [22]. As mentioned above, the conditions for the stable growth of crystals must be Q_1_ > 0, that is, there must be a positive temperature gradient during the crystal growth process. The importance of the thermal field and temperature gradient to crystal growth can thus be seen.

According to our previous reports, for the radio frequency heating AlN crystal growth furnace, when the crucible is at a relative position of 16%, the radial temperature gradient at the seed is about 4.6 K/cm, and the axial temperature gradient from the seed to the material surface is about 7.29 K/cm [13]. The temperature gradient reaches a maximum value at this time. According to the actual growth results, the large radial temperature gradient at this position is beneficial to the diameter expansion of the small seed. Therefore, we want to adjust the thermal field of the AlN crystal growth resistance furnace to the most optimal status. If a diameter expansion growth process for a small seed is realized, a slightly larger radial temperature gradient has to be used. If a large-sized seed crystal is used for growth in the later stage, the radial temperature gradient can be adjusted to be small. This work mainly simulates the following heating modes: (1) side heater 1 as model 1, (2) side 1 heater + bottom heater as model 2, (3) side 1 heater + top heater as model 3, and (4) side 1 heater + side 2 heater as model 4.

**Figure 2:**
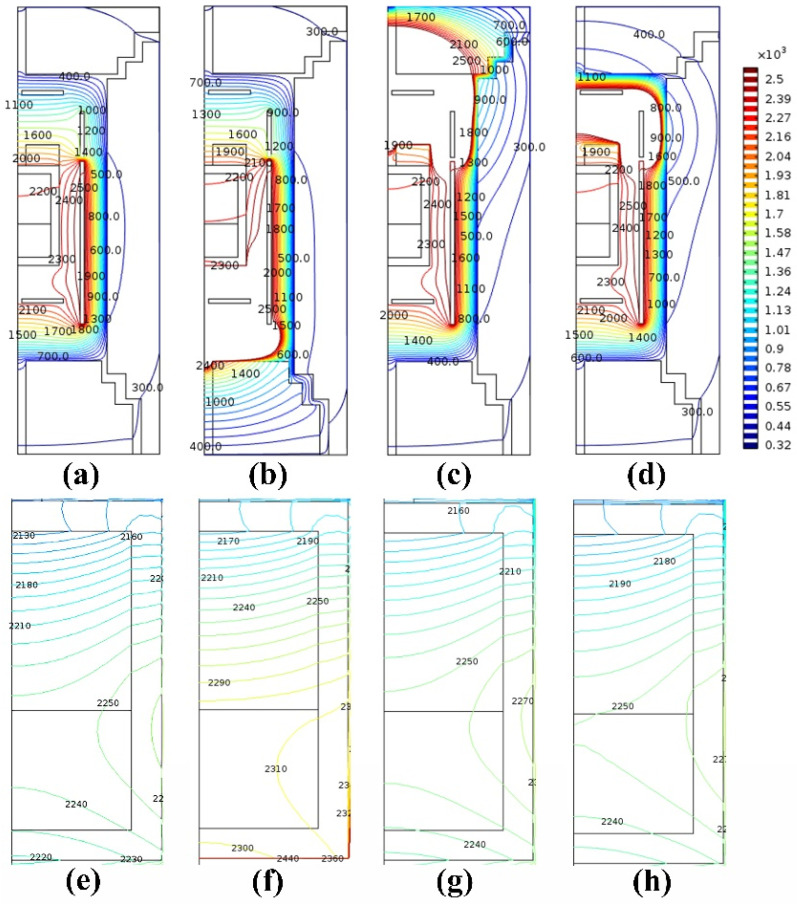
The distribution of temperature in the growth chamber and inside the crucible with different heating modes: (**a**,**e**) side heating 1 mode, (**b**,**f**) side 1 heater + bottom heater mode, (**c**,**g**) side 1 heater + top heater mode, and (**d**,**h**) side 1 heater + side 2 heater mode.

Figure 2 shows the temperature distribution of the growth chamber and the crucible under different segmented heating modes calculated by simulation. It can be seen from Figure 2a–d that when the auxiliary heater is added, the temperature at the corresponding position of the auxiliary heater will increase accordingly. For example, by adding a bottom heater, the overall temperature at the bottom rises greatly, and the highest temperature at the bottom of the crucible reaches 2310 °C. When the top heater is added, the overall temperature of the top rises greatly. When the temperature at the seed position is the same, the inner temperature of the upper insulation increases from 700 °C to about 2000 °C.

The effect of adding the auxiliary side heater 2 is similar to the effect of adding the top heater. With the addition of the bottom heater, the axial temperature gradient tends to increase. The isotherms at the surface of the raw material and the growth cavity are relatively smooth. In the case of other heating modes, there is a vertical isotherm at the junction of the surface of the raw material and the growth area of the crucible, as shown in Figure 2e,f.

**Figure 3:**
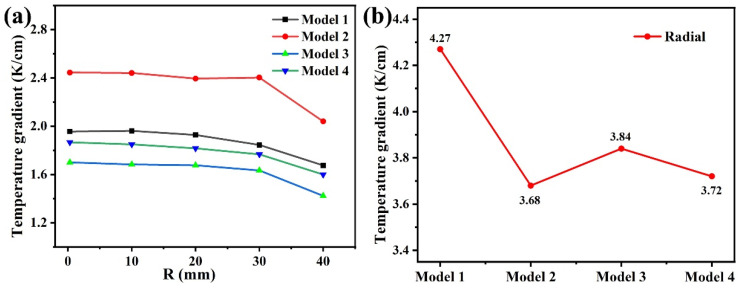
(**a**) The temperature gradient change in the Z direction at different positions along the R direction in different heating modes. (**b**) Radial temperature gradient change under different heating modes.

Figure S1 is a schematic diagram of the Z direction at different positions along the R direction. R represents the radial direction, z represents the axial direction, the central position R is 0, and the position R close to the crucible wall is 40 mm. Figure 3a shows the temperature gradient change in the Z direction at different positions along the R direction in different heating modes, and the statistical data are shown in Table S1. According to the analysis of Table S1, the minimum axial temperature gradient is 1.7 K/cm (R = 0.3 mm) at the side 1 heater + the top heater mode, and the maximum axial temperature gradient is 2.445 K/cm (R = 0.3 mm), which corresponds to the side 1 heater + bottom heater mode. Figure 3 is the statistical curve of Table S1; it describes the temperature gradient of several modes in the Z direction at different positions along the R direction. From the analysis of Figure 3a, it can be seen that the temperature gradient in the Z direction at different positions of the R direction for the side 1 heater + bottom heater mode is the largest, and the temperature gradient of the side 1 heater + top heater is the smallest. The temperature gradients of the other two models are at a moderate level, and it can be analyzed that the temperature near the wall of the growth chamber is higher.

The axial temperature gradients of the remaining two models are somewhere in between. However, the axial temperature gradient for the side 1 heater + side 2 heater is smaller than that for the side heater 1 mode. Through analysis, it can be concluded that the top heater has a significant effect on reducing the axial temperature gradient. We speculate that if the heating power of the top heater is increased, the axial temperature gradient at the seed position may be reversed, and the problem of the multi-point nucleation of AlN crystals at the early stage of crystal growth may be suppressed. The influence of the side heater 2 on the axial temperature gradient is similar to that of the top heater, but the effect is slightly weaker. The bottom heater causes the increase of the axial temperature gradient, which can significantly increase the temperature of the bottom of the crucible and increase the temperature of the raw materials. According to the results in Figure 3b, it is found that the radial temperature gradient for the side heater 1 mode is the largest (4.27 K/cm), the radial temperature gradients for the other three heating modes are basically the same, and the radial temperature gradient for the side 1 heater + bottom heater mode is the smallest (3.68 K/cm). Based on the simulation results, it can be seen that the radial temperature gradient required for the diameter expansion of the small seed can be obtained by these several simulation heating modes. However, the adjustable range of the axial temperature gradient of the side 1 heater + the bottom heater is the largest.

The axial temperature gradient determines the growth rate of the crystal. It is the driving force for crystal growth. There is a linear relationship between the growth rate and the axial temperature gradient. A larger radial temperature gradient is beneficial to reduce the nucleation density and enlarge the crystal size during the initial growth process, and has a greater impact on the nucleation and morphology of the crystal [13,14]. Model 1 has the largest radial gradient (4.27 K/cm), and the axial temperature gradient is moderate (1.96 K/cm). Therefore, we believe that model 1 is suitable for the expansion and growth of small-size crystals. Model 2 has the smallest radial gradient (3.68 K/cm) and the largest axial temperature gradient (2.445 K/cm), which is suitable for the growth of large-size crystals. Model 3 has a moderate radial gradient (3.84 K/cm) and minimum axial temperature gradient (1.7 K/cm), which is suitable for the growth of high-quality crystals. Model 4 has a moderate radial gradient (3.72 K/cm) and an axial temperature gradient of 1.87 K/cm. Similar to model 3, it is suitable for the growth of large-size and high-quality crystals.

## 4. Summary

In this paper, the thermal field of a self-designed AlN crystal growth resistance furnace under different heater structures was simulated and the following conclusions were drawn: (1) the top heater can effectively reduce the axial temperature gradient, and the side heater 2 has a similar effect on the axial gradient as the top heater, but the effect feedback is slightly weaker. The bottom heater can increase the axial temperature gradient. It obviously increases the temperature of the bottom of the crucible and the raw materials. (2) The side heater 1 mode has the largest radial temperature gradient, and the other three models have moderate radial temperature gradients, which can meet the requirement of crystal growth. (3) The axial temperature gradient tends to increase when the bottom heater is added to the furnace, and the isotherm at the junction of the raw material surface and the crystal growth chamber is relatively smooth. With the combination of other heaters, there is a vertical isotherm at the junction of the raw material surface and the growth chamber.

## Figures and Tables

**Table 1:** Physical properties of the materials.

	AlN	W	Mo	Al	Stainless Steel
Thermal conductivity, k (W m^−1^k^−1^)	220	175	138	138	44.5
Isobaric specific heat, C_p_ (J kg^−1^K^−1^)	1197	132	250	900	475
Density, ρ (kg m^−3^)	2702	17,800	10,200	2700	7850
Emissivity, ε	0.08	0.04	0.08	0.07	0.85

## Supplementary Materials

The following are available online at https://www.mdpi.com/article/10.3390/ma14237441/s1, Figure S1: Schematic diagram of the Z direction at different positions along the R direction, Table S1: The temperature gradient along the Z direction at different positions along the R direction.
