# Supplementary material for: Influence of Different Heater Structures on the Temperature Field of AlN Crystal Growth by Resistance Heating
Source: Materials (Basel). 2021 Dec 4;14(23):7441. doi: 10.3390/ma14237441 (PMC8658778; doi:10.3390/ma14237441)
Supplement: Supplementary file 1 [file materials-14-07441-s001.zip › materials-1426462-supplementary.pdf]

materials

Article

Influence of Different Heater Structures on the Temperature Field of AlN Crystal Growth by Resistance Heating

Ruixian Yu [1], Chengmin Chen [2,3], Guodong Wang [1,*], Guangxia Liu [2], Shouzhi Wang [1], Xiaobo Hu [1,*], Ma Lei [4], Xiangang Xu [1] and Lei Zhang [1,*]

[1] State Key Laboratory of Crystal Materials, Institute of Novel Semiconductors, Shandong University, Jinan 250100, China; yuruixian0001@126.com (R.Y.); wangshouzhi6@163.com (S.W.); xxu@sdu.edu.cn (X.X.)
[2] Energy Institute, Qilu University of Technology (Shandong Academy of Sciences), Jinan 250100, China; cm_chen1989@163.com (C.C.); liugx@sderi.cn (G.L.)
[3] Jinan Institute of supercomputing technology, Jinan 250100, China
[4] School of Opto-Electronic Engineering, Zaozhuang University, Zaozhuang 277160, China; leima_1017@163.com
* Correspondence: guodong9631@sdu.edu.cn (G.W.); xbhu@sdu.edu.cn (X.H.); leizhang528@sdu.edu.cn (L.Z.)

Citation: Yu, R.; Chen, C.; Wang, G.; Liu, G.; Wang, S.; Hu, X.; Lei, M.; Xu, X.; Zhang, L. Influence of Different Heater Structures on the Temperature Field of AlN Crystal Growth by Resistance Heating. *Materials* **2021**, *14*, 7441. https://doi.org/10.3390/ ma14237441

Academic Editor: Rainer Niewa

Received: 1 October 2021
Accepted: 30 November 2021
Published: 4 December 2021

Publisher's Note: MDPI stays neutral with regard to jurisdictional claims in published maps and institutional affiliations.

Copyright: © 2021 by the authors. Licensee MDPI, Basel, Switzerland. This article is an open access article distributed under the terms and conditions of the Creative Commons Attribution (CC BY) license (https://creativecommons.org/licenses/by/4.0/).

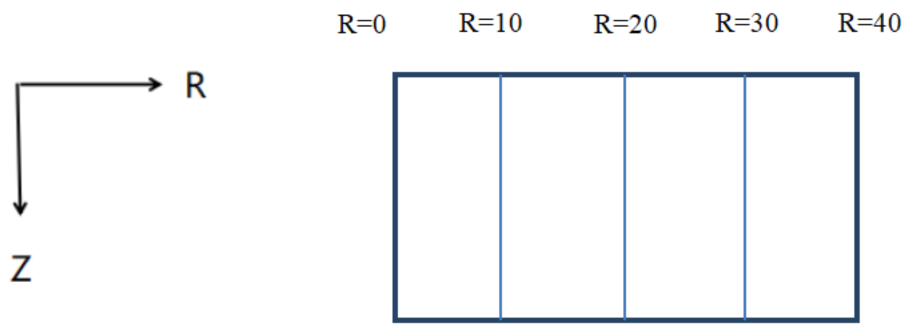

Figure S1. Schematic diagram of the Z direction at different positions along the R direction.

Table S1. The temperature gradient along the Z direction at different positions along the R direction (The unit is K/cm, 0.3 mm is the position close to the center, and 40 mm is the position close to the crucible wall）.

R position	Side heating 1	Side 1 heater + bottom heater	Side 1 heater + top heater	Side 1 heater + side 2 heater
0.3	1.956667	2.445	1.7	1.866666667
10	1.961667	2.441666667	1.683333333	1.85
20	1.928333	2.395	1.676666667	1.816666667
30	1.845	2.403333333	1.633333333	1.766666667
40	1.675	2.04	1.423333333	1.6
